# Differences in Brain Functional Networks of Executive Function Between Cantonese-Mandarin Bilinguals and Mandarin Monolinguals

**DOI:** 10.3389/fnhum.2021.748919

**Published:** 2021-11-18

**Authors:** Lei Cai, Xiaoyu Xu, Xiaoxuan Fan, Jingwen Ma, Miao Fan, Qingxiong Wang, Yujia Wu, Ning Pan, Zhixin Yin, Xiuhong Li

**Affiliations:** ^1^Department of Maternal and Child Health, School of Public Health, Sun Yat-sen University, Guangzhou, China; ^2^Wuhan Children’s Hospital, Tongji Medical College, Huazhong University of Science and Technology, Wuhan, China; ^3^Guangdong Provincial Maternal and Child Health Care Hospital, Guangzhou, China; ^4^The First Affiliated Hospital of Sun Yat-sen University, Guangzhou, China

**Keywords:** bilingualism, executive function, network-based statistics, functional connectivity, resting-state functional connectivity magnetic resonance imaging (R-fMRI)

## Abstract

It remains controversial whether long-term logographic-logographic bilingual experience shapes the special brain functional subnetworks underlying different components of executive function (EF). To address this question, this study explored the differences in the functional connections underlying EF between the Cantonese-Mandarin bilinguals and Mandarin monolinguals. 31 Cantonese-Mandarin bilinguals and 31 Mandarin monolinguals were scanned in a 3-T magnetic resonance scanner at rest. 4 kinds of behavioral tasks of EF were tested. Network-based statistics (NBS) was performed to compare the connectomes of fronto-parietal (FP) and cingulo-opercular (CO) network between groups. The results showed that the bilinguals had stronger connectivity than monolinguals in a subnetwork located in the CO network rather than the FP network. The identified differential subnetwork referred to as the CO subnetwork contained 9 nodes and 10 edges, in which the center node was the left mid-insula with a degree centrality of 5. The functional connectivity of the CO subnetwork was significantly negatively correlated with interference effect in bilinguals. The results suggested that long-term Cantonese-Mandarin bilingual experience was associated with stronger functional connectivity underlying inhibitory control in the CO subnetwork.

## Introduction

With the development of globalization, more than half of the world’s population is bilingual (Grosjean, 2013). It is well known that when bilinguals use one language, the non-target language is jointly activated (Kroll et al., 2014, 2015). According to the inhibitory control model (Green, 1998) and adaptive control hypothesis (Green and Abutalebi, 2013), the non-target language is inhibited so that the target language could be processed appropriately. Therefore, it is believed that long-term bilingualism experience may shape the brain functional network underlying executive function (EF).

Emerging brain functional neuroimaging studies have attempted to reveal the neural substrates of bilingualism’s effect on EF. Most of these studies concentrated on which brain regions were activated in bilinguals during certain EF tasks (see reviews (Pliatsikas and Luk, 2016; Hayakawa and Marian, 2019)). However, the term EF includes a series of abilities, such as response inhibition, interference inhibition, set-shifting between task sets, updating, planning, and monitoring (Friedman and Miyake, 2017). Because different EF tasks were used across functional magnetic resonance imaging (fMRI) studies on bilingualism, different brain regions associated with EF were identified in these studies. Specifically, cingulate gyrus (Abutalebi et al., 2012; Mohades et al., 2014), temporal gyrus (Mohades et al., 2014), basal ganglia (Stocco et al., 2014), thalamus (Yoon, 2011) and the left inferior parietal lobule (Ansaldo et al., 2015) were found to get involved in inhibition control related tasks. Prefrontal cortex and caudate nucleus were reported to participate in switching tasks (Rodriguez-Pujadas et al., 2013). The anterior cingulate cortex, basal ganglia and prefrontal lobe were deemed as the core cognitive control regions for bilinguals (Seo et al., 2018). Resting-state fMRI provides a measure of coordination (time-course correlation) that reflects a broad history of coactivity across many tasks rather than relying on relationships defined in a single task (Vogel et al., 2010; Laumann et al., 2017). However, studies using resting-state fMRI to investigate the brain networks underlying EF in bilinguals are limited (Grady et al., 2015; Berken et al., 2016; Kousaie et al., 2017). Two studies (Berken et al., 2016; Kousaie et al., 2017) used a seed map analysis to analyze functional connectivity related to EF in bilinguals, and they used EF-related regions as the seeds, the left inferior frontal gyrus (lIFG) and ventral medial prefrontal cortex (vmPFC), respectively. The seed map analysis was limited by the a priori selection of seed regions, so the two studies identified different EF networks.

The literature has shown that EF requires the cooperation of multiple brain areas, not just independent activities of one or several brain areas. According to the dual-network model (Dosenbach et al., 2008; Gratton et al., 2018), EF could be attributed to two separated but strongly interconnected brain networks: the fronto-parietal (FP) network and the cingulo-opercular (CO) network (also called the salience network, SN). The FP network includes dorsolateral and inferior frontal regions, and the inferior parietal lobes act to initiate and adjust control (Cole and Schneider, 2007). The CO network, including the anterior insula (AI), basal ganglia and dorsal anterior cingulate cortex (dACC) stretching into the medial frontal cortex, is considered a core system involved in self-regulation, resolution of conflicts between competing information, and focal attention (Dosenbach et al., 2008). Recent research also suggested that both the CO and FP networks are associated with working memory (Wallis et al., 2015; Assem et al., 2020). The core structures in both the CO and FP networks were reported to get involved in bilinguals’ EF by previous task-based fMRI studies, as mentioned above. Previous studies on white matter also reported the structure of white matter tracts connecting frontal lobe, thalamus and basal ganglia were related to executive control in bilinguals (Mamiya et al., 2018; Macbeth et al., 2021). However, thus far, it remains unclear which EF brain network is affected by long-term bilingual experience. Grady et al. (2015) found that there was stronger connectivity in the FP network but not the CO network in life-long bilingual older adults than monolinguals. The study suggested that life-long bilingual experience influences specific brain networks related to EF.

Some studies have shown that when considering the influence of bilingualism on the brain network underlying EF, language similarity between the two languages should not be ignored (Wichmann et al., 2010; Lehtonen et al., 2018). Greater language similarity between the first language (L1) and the second (L2) may lead to stronger cross-language interference, which requires stronger EF to inhibit interference. For example, Coderre and van Heuven (2014) compared EF among three different bilingual groups whose orthographic similarity ranged from high to low, including German-English, Polish-English and Arabic-English. They found that compared to Polish-English and Arabic-English bilinguals, German-English bilinguals with the highest similarity in orthography had the best EF abilities. However, it’s still unclear whether the bilingualism with the highest similarity in orthography shapes the special brain functional networks underlying EF.

In China, Mandarin is the official language, while Cantonese is a local native language in Guangdong Province. Cantonese-Mandarin bilinguals use Cantonese in daily oral communication while using Mandarin as their official and written language. Cantonese-Mandarin bilinguals are unique subjects who are proficient logographic-logographic bilinguals, and the main difference between Cantonese and Mandarin in mainland China lies in phonology, but they shared the same writing system. Therefore, compared to general bilinguals, Cantonese-Mandarin bilinguals may need stronger EF to inhibit interference of the non-target language, which may lead to reconfigurations in the brain network relevant to EF. There were more than 1 billion users of Mandarin and 62 million users of Cantonese in China^1^. Investigating the neurocognitive consequences of Cantonese-Mandarin bilinguals would help to understand the mechanisms underlying the promotion of EF driven by bilingualism, which could subsequently influence educational and public policy decisions regarding language inclusivity in schools.

In this study, we aimed to examine whether long-term logographic-logographic experience could shape special brain networks underlying different components of EF. We used network-based statistics (NBS) in the analysis of resting-state fMRI data to investigate whether there was different functional connectivity in the CO network and FP network between Cantonese-Mandarin bilinguals and Mandarin monolinguals. We further examined the correlation between the behavioral tasks of EF and the mean functional connectivity in the identified subnetworks with group-wise differences.

## Materials and Methods

### Participants

Sixty-two participants were recruited from colleges in Guangzhou, China. 31 participants were Cantonese-Mandarin bilingual speakers who learned Mandarin as their L2 after age 3 (bilingual group: *N* = 31, 24 females and 7 males; age = 21.1 ± 2.0 years). 31 participants were native Mandarin speakers who did not learn any other Chinese (monolingual group: *N* = 31, 22 females and 9 males; age = 21.5 ± 2.0 years). All the participants reported that they had normal vision or corrected-to-normal vision, audition, and mental health. None of them had nerve injuries or had taken psychotropic drugs in the past 6 months. A self-rating scale was used to evaluate the proficiency of speaking, comprehension, reading and writing of Cantonese and Mandarin, where 10 and 0 indicated the most and least proficient, respectively. The self-rating scale was a Chinese translation of the Language and Social Background Questionnaire (LBSQ) developed by York University, which has been proven to be reliable and valid and appropriate for non-English language assessment (Anderson et al., 2018). As English was a compulsory course in Chinese, we also collected the information of their levels in national standardized English tests, College English Test (CET) to evaluate their English proficiency.

Raven’s Standard Progressive Matrices Test was performed to assess intelligence quotient (IQ). No significant difference was observed in age, IQ, gender distribution or any indices of socioeconomic status between the two groups (see Table 1). All participants were right-handed as diagnosed with a handedness inventory (Oldfield, 1971; Li, 1983). The subjects did not have attention deficit hyperactivity disorder, learning disabilities, neurological diseases, psychiatric disorders, or visual or hearing difficulties.

**TABLE 1:** Demographic data of participants.

Variables	Bilinguals (*N* = 31)	Monolinguals (*N* = 31)	*T*/*U*/χ^2^	*P*
Age (*M* ± *SD*, years)	21.10 ± 1.97	21.40 ± 2.03	425.00	0.428
Gender (female/male, *N*)	24/7	22/9	0.34	0.562
IQ score (*M* ± *SD*)	122.68 ± 11.76	124.33 ± 14.16	–0.50	0.621
**Socio-economic status (*N*)**				
Education (undergraduate/postgraduate)	23/8	23/8	0	1.000
District of residence (city/suburb/missing)	20/10/1	20/10/1	0	1.000
Father’s education			0.11	0.948
Junior high school or below	13	12		
Senior high school or technical secondary school	7	8		
College or above	11	11		
Mother’s education			1.15	0.562
Junior high school or below	11	14		
Senior high school or technical secondary school	7	8		
College or above	13	9		
**Language proficiency (*M* ± *SD*)**				
Speaking in Mandarin	8.55 ± 1.15	8.87 ± 1.20	383.50	0.222
Speaking in Cantonese	9.42 ± 0.77	–	–	–
Reading in Mandarin	8.90 ± 0.98	9.03 ± 0.93	430.00	0.591
Reading in Cantonese	7.77 ± 1.41	–	–	–
Writing in Mandarin	8.61 ± 1.15	8.77 ± 1.28	423.00	0.529
Writing in Cantonese	5.74 ± 2.10	–	–	–
Comprehension of Mandarin	8.84 ± 1.10	8.77 ± 1.17	446.50	0.778
Comprehension of Cantonese	8.87 ± 0.92	–	–	–
English level (band 4/band 6, *N*)	15/16	13/18	0.26	0.610

*Chi-square test was used for comparison of gender distribution, and independent-samples T-test and Mann–Whitney U test was separately used for comparison of the normal and non-normal distributed variables. The χ^2^value of the comparison of gender, Socio-economic status and English level, the T score of the comparisons of the IQ score, and the U score of the comparisons of the rest variables were displayed in the table.*

Written informed consent was obtained from all the subjects. The research protocol and content were approved by the committee and were in accordance with ethical standards of the Medical Ethics Committee, Sun Yat-sen University with the ethical approval number [L2016] No.036.

### Behavioral Tasks of Executive Function

Executive function include three components: inhibitory control, shift, and working memory (Friedman and Miyake, 2017). Furthermore, inhibitory control contains interference control and response inhibition.

In the current study, Stroop color and word test was used to measure the ability of interference control (Wang et al., 2017). Four colored Chinese characters, “
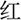
”, “
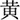
”, “
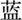
”, “
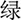
”, which means red, yellow, blue, green, respectively, were randomly presented in the screen. Subjects were asked to identify the color of the font presented by pressing the corresponding key. The response interval duration was not limited. There were three conditions in the Stroop task. In the first condition, the colors were matched to meaning for the presented fonts (congruent). In the second condition, the colors and meanings were unmatched for the presented fonts (incongruent). The third one was the neutral condition, in which a colored “X” was presented. The trials of the three conditions were performed randomly. The interference effect in Stroop test was calculated according to the formula (Scarpina and Tagini, 2017): total time + [(2 × mean time per word) × number of uncorrected errors]. The higher the interference effect was, the poorer ability of interference control.

Go/no-go paradigm was adopted to measure the response inhibition. Subjects were presented with the letters of “F” or “J” and told to press the corresponding keys, but not to respond when a red dot appeared above the letters, namely a no-go signal (Verbruggen and Logan, 2008). The response interval duration was set at 1,000 ms. The response inhibition score was calculated by the mean RT of correct responses.

The color-shape switch paradigm was used to measure the ability of shift. The stimulus contains two kinds of shapes in two colors (Karbach and Kray, 2009). The first two blocks used single tasks, in which subjects were asked to judge either the color or shape of the stimulus by press the corresponding keys. The third block used mixed tasks, in which the subjects were not told the requirement in advance, and they judged the characters of stimulus according to the hint words (color or shape) on every trial. The response interval duration was set at 1,000 ms. The shift score was calculated by the mean RT of correct responses.

The above paradigms measuring inhibitory control and shift were compelled by eprime2.0, and the detailed design was reported in the Supplementary Figures 1–3.

The working memory ability was measured by the score of digit span test in the Wechsler-IV Adult intelligence Scale-Revised Chinese version (WAIS-SR). The test was performed in Cantonese for the Cantonese-Mandarin bilinguals, and Mandarin for the Mandarin monolinguals.

### Resting-State Functional Magnetic Resonance Imaging Data Acquisition

The MR imaging was performed using a 3T scanner (Siemens Magnetom TrioTim syngo) at the Brain Imaging Center of Institute for Brain Research and Rehabilitation, South China Normal University. Functional images were collected with an echo-planar imaging sequence using the following parameters: TR = 2,000 ms, TE = 30 ms, matrix size = 64 × 64, field of view (FOV) = 224 mm × 224 mm, flip angle = 90°, slice thickness = 3.5 mm, 32 interleaved slices, and 240 volumes acquired in 8 min. The participants were instructed to lie down, keep their eyes open, not think about anything specific, and remain still.

T1-weighted 3D images were collected with the following parameters: TR = 1,900 ms, TE = 2.52 ms, matrix size = 256 × 256, FOV = 256 mm × 256 mm, flip angle = 9°, slice thickness = 1.00 mm, and 176 slices covering the whole brain.

### Resting-State Functional MRI Data Preprocessing

The functional images were processed using the Data Processing Assistant for Resting-State fMRI tool [DPARSF^2^ (Yan and Zang, 2010)], which is based on Statistical Parameter Mapping (SPM^3^) and the toolbox for Data Processing and Analysis of Brain Imaging (DPABI^4^ (Yan et al., 2016)). The first 10 volumes were discarded to reduce the influence of the unstable initial MR imaging signal. Then, slice timing correction and realignment for head motion correction were performed. The individual T1-weighted images were registered to the mean functional images. A unified segmentation algorithm was then used to segment the transformed structural images into gray matter, white matter, and cerebrospinal fluid. To control for the possible effects of head motion and the global white matter and cerebrospinal fluid signals on the results, we regressed out the nuisance covariates (head motion profiles derived from the Friston 24-parameter model, white matter signal, and cerebrospinal fluid signal) within each voxel in the whole brain. Then, based on the normalization parameters estimated during unified segmentation, the motion-corrected functional images were further normalized to the standard Montreal Neurological Institute (MNI) space and resampled to 3 mm × 3 mm × 3 mm. Subsequently, the functional images were spatially smoothed using a Gaussian kernel with 4-mm full width at half maximum. The resulting data were further bandpass-filtered (0.01–0.1 Hz) to reduce the low-frequency drift and high-frequency physiological respiratory and cardiac noise. Because regressing out the global signal in resting-state fMRI analysis mandates the presence of anti-correlations (Murphy and Fox, 2017) and can have differential effects on the groups, we did not regress out the global signal in this study. All 62 subjects were included in further analysis following the excluding standard (Yan et al., 2013) based on motion [mean frame-wise displacement (FD); Jenkinson et al. (2002)] greater than 2*SD above the group mean motion (threshold: 0.192). In addition, no difference in the mean FD was found between the two groups (bilingual group: mean FD = 0.061 ± 0.025, monolingual group: mean FD = 0.070 ± 0.032, *P* = 0.241).

### Construction of Functional Brain Networks

All networks are composed by the basic elements, so-called nodes, and the pairwise relationships, so-called edges. In functional brain networks, nodes represent pre-defined portions of brain tissue, and edges represent the functional connectivity between pairs of nodes (Matthew et al., 2013). We used the advanced edition of DPARSF4.4^5^ for network reconstruction. To obtain a functional connectivity matrix of cognitive control for each subject, we defined a cingulo-opercular (CO) network of 32 nodes and a frontal-parietal (FP) network of 21 nodes based on the Dosenbach template (Dosenbach et al., 2010). The defined CO network contains 3 nodes in cingulate cortex, 6 nodes in frontal lobe, 6 nodes in insula, 3 nodes in thalamus and the rest nodes are located in basal ganglia, temporal and parietal lobes. The FP network contains 12 nodes in frontal lobe, 8 nodes in parietal lobe and 1 in anterior cingulate cortex. The detailed regions and MNI coordinates of the selected nodes were presented in Supplementary Table 1. Each selected node was set to be a 5 mm radius sphere and none of the nodes overlapped. For a given node region, the mean time course was calculated as the average of the fMRI time series from all voxels within that region. Then, the correlation matrices were obtained by computing the Pearson correlation coefficient between the mean time course of each pair of nodes. Because of the uncertain meaning of negative connectivity, we included only positive connectivity in all our analyses (Murphy et al., 2009; Chai et al., 2012). Specifically, the negative correlations were converted to zero. We computed the average function connectivity of the 53 × 53 (32 + 21 nodes) functional connectivity matrix, and detected an outlier more than three quartiles away from *P*_75_ in the monolingual group (see the boxplot, Supplementary Figure 4). We removed the outlier before further analysis.

### Network-Based Statistics

For the obtained functional connectivity matrix, we performed the NBS analysis in GRETNA software (Wang et al., 2015). NBS Zalesky et al. (2010) was used for the statistical comparison. Compared to alternative methods for analyzing resting-state fMRI data (such as the seed-based correlation approach and independent component analysis, ICA), NBS considers the whole brain as an integrated system (a connected network) rather than a collection of individual components (Bullmore and Sporns, 2009). Notably, NBS controls for multiple comparisons through cluster-based threshold, in which connected components of a network are considered as a cluster. NBS can yield substantially greater statistical power than generic procedures if any connected component (subnetwork) in which the connections show significant between-group differences exist. In other words, NBS could be more sensitive in identifying which subnetwork, if any, exhibits significant functional differences between groups. In this study, to compare the functional connectivity matrices of the two groups, a two-sample *t*-test was used with 5,000 permutations, and the significance level *P* < 0.05 was corrected for multiple comparisons using NBS correction. The head motion parameter (mean FD; Jenkinson) and gender were included as a covariate.

Since NBS results are highly dependent on the initial cluster-defining threshold although no standard value has been established, the threshold selection is arbitrary. We performed an additional series of NBS analyses with distinct initial cluster-defining thresholds (*t* = 2.0–3.5) as described in previous studies (Smith et al., 2013; Gaudio et al., 2018; Pascual-Belda et al., 2018). As GRETNA software does not allow to set *t* threshold, but edge *P*-values, we tested different edge *P* setting from 0.05 to 0.001 (see Table 3). Only the most significant results of the NBS analysis with a cluster-defining threshold (*t* = 2.9, edge *P* = 0.005) will be shown in this article. The identified brain network was visualized with BrainNet Viewer^6^ (Xia et al., 2013).

**TABLE 2:** The behavioral performance of executive function (EF) in two groups.

EF	Bilinguals (*N* = 28)	Monolinguals (*N* = 25)	*P*
**Inhibitory control (*M* ± *SD*)**			
Interference control (ms)^a^	115801.92 ± 19383.86	116551.66 ± 31994.68	0.918
Response control (ms)^b^	308.11 ± 31.13	309.07 ± 35.16	0.914
Shift (ms)^b^ (*M* ± *SD*)	610.35 ± 101.09	647.07 ± 144.02	0.288
Working memory^c^ (*M* ± *SD*)	33.75 ± 3.09	32.56 ± 4.24	0.250

*Gender was controlled as a covariate.*

*^a^The interference control was measured by the RT and the correct rate in the Stroop paradigm by the formula: total time + [(2 × mean time per word) number of uncorrected errors], and the higher value indicated the weaker interference control ability.*

*^b^The response inhibition and shift were measured by the mean RT of correct responses, and the higher value indicated the weaker ability.*

*^c^The working memory ability was measured by the score of digit span test in the Wechsler-IV intelligence test and the higher value indicted stronger working memory.*

**TABLE 3:** Network-based statistics results in the cingulo-opercular network at different primary thresholds.

Threshold	Edge *P*	Nodes	Edges	*P*
2	0.05	26	60	0.022
2.4	0.02	20	28	0.028
2.7	0.01	15	18	0.017
2.9	0.005	9	10	0.016
3.2	0.002	3	3	0.034
3.5	0.001	4	2	0.032

### Correlation Analysis

R4.0.2 was used for the following statistical analyses. First the strength of the functional connectivity within the subnetworks identified by the NBS analysis was calculated. Considering the non-normal distribution of the original functional connectivity (Pearson correlation coefficient), here, we use Fisher’s transformed *Z* to calculate the mean functional connectivity within the identified subnetworks. Then, an interaction analysis was conducted to compare the correlation of functional connectivity with behavior scores between the two groups. Partial correlation analysis was used to assess the association between inhibitory control (including interference control and response inhibition), shift abilities and working memory with functional connectivity, after controlling the effect of gender. If the interaction effect of the group was significant (*P*_interaction_ < 0.05), the partial correlation would be performed in the subgroup, otherwise it would be performed in the whole sample. Bonferroni correction was performed for multiple statistical tests.

## Results

### Sample Characteristics

Table 1 shows the demographics of the bilingual and monolingual groups. No significant difference in the proficiency of Mandarin and English was found between the groups. There was no significant difference between the groups in age, gender, IQ, and indices of socioeconomic status (all *P* > 0.05).

### Behavioral Performance of Executive Function

Analysis of covariance was performed for group-wise comparison after controlling gender. As shown in Table 2, there was no significant difference between the two groups in the performance of these EFs (interference control: 115801.9 ± 19.4 vs. 116551.7 ± 32.0, *P* = 0.918; response inhibition: 308.1 ± 31.1 vs. 309.1 ± 35.2, *P* = 0.914; shift: 610.4 ± 101.1 vs. 647.1 ± 144.0, *P* = 0.288; working memory: 33.8 ± 3.1 vs. 32.6 ± 4.2, *P* = 0.250).

### Subnetwork Identified by Network-Based Statistics Analysis

**FIGURE 1:**
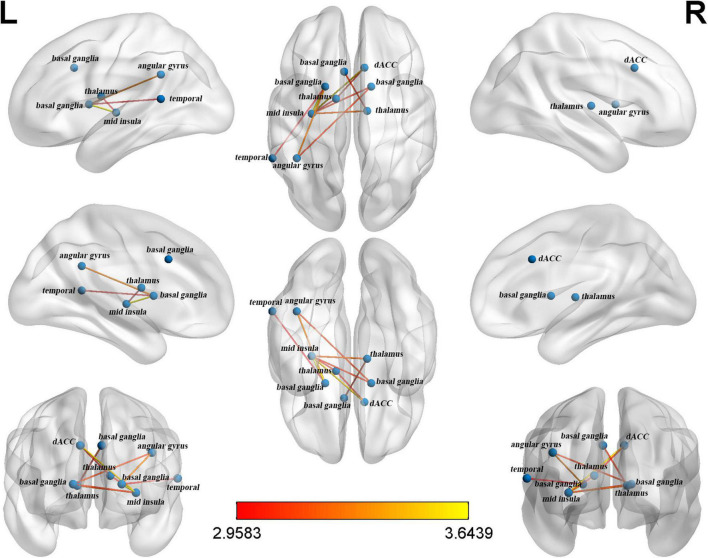
The cingulo-opercular (CO) subnetwork identified by network-based statistics.

A significant subnetwork between the two groups was found only in the CO network and not in the FP network. To identify the subnetwork exhibiting group-wise differences within the CO network, NBS was performed with different primary thresholds in the range between 2.0 and 3.5 (Table 3). The most significant difference between the groups was observed at a threshold of *t* = 2.9 (*P* = 0.016, two-sided). The resulting subnetwork comprised 9 nodes: right dACC, left basal ganglia (2 nodes refer to it), right basal ganglia, left thalamus, right thalamus, left mid-insula, left temporal and left angular gyrus. This subnetwork included 10 connections (Table 4). Figure 1 shows a view of this network obtained with the BrainNet Viewer toolbox. This subnetwork mainly includes regions pertaining to the core areas of the CO network. In the subnetwork, the left mid-insula demonstrated the most degree centrality with a value of 5, which suggested a possibly crucial role of the left mid-insula in the CO subnetwork. The degree centrality of the left basal ganglia was 3. The degree centrality of the right dACC, left thalamus, right basal ganglia and left angular gyrus were all 2. The left temporal had only one connection with the angular gyrus.

**TABLE 4:** Functional connectivity within the subnetwork identified by network-based statistics (NBS) analysis in the cingulo-opercular network.

Node 1	MNI coordinate	Node 2	MNI coordinate	Functional connectivity (Fisher *Z* transformed, *M*(*SD*)]		*F*	*P*
				Bilinguals	Monolinguals		
dACC (R)	(9, 20, 34)	Thalamus (L)	(−12, 3, 13)	0.45 (0.15)	0.32 (0.16)	9.134	0.004
dACC (R)	(9, 20, 34)	Mid-insula (L)	(−30, 14, 1)	0.49 (0.14)	0.38 (0.13)	12.356	<0.001
Basal ganglia (L)	(−6, 17, 34)	Thalamus (R)	(11, 12, 6)	0.21 (0.17)	0.09 (0.11)	10.306	0.002
Basal ganglia (L)	(−20, 6, 7)	Mid-insula (L)	(−30, 14, 1)	0.57 (0.18)	0.43 (0.18)	13.289	<0.001
Basal ganglia (L)	(−20, 6, 7)	Angular gyrus (L)	(−41, 47, 29)	0.26 (0.14)	0.15 (0.12)	9.910	0.003
Basal ganglia (L)	(−20, 6, 7)	Temporal (L)	(−59, −47, 11)	0.60 (0.17)	0.47 (0.19)	7.527	0.008
Basal ganglia (R)	(14, 6, 7)	Mid-insula (L)	(−30, 14, 1)	0.52 (0.20)	0.37 (0.17)	9.181	0.004
Basal ganglia (R)	(14, 6, 7)	Angular gyrus (L)	(−41, 47, 29)	0.45 (0.15)	0.32 (0.16)	10.825	0.002
Thalamus (L)	(−12, 3, 13)	Mid-insula (L)	(−30, 14, 1)	0.39 (0.17)	0.25 (0.14)	8.681	0.005
Thalamus (R)	(11, 12, 6)	Mid-insula (L)	(−30, 14, 1)	0.64 (0.18)	0.49 (0.14)	10.009	0.002

*dACC, dorsal anterior cingulate cortex; vFC, ventral frontal cortex; R, right; L, left.*

### Group Differences in Identified Subnetwork

**FIGURE 2:**
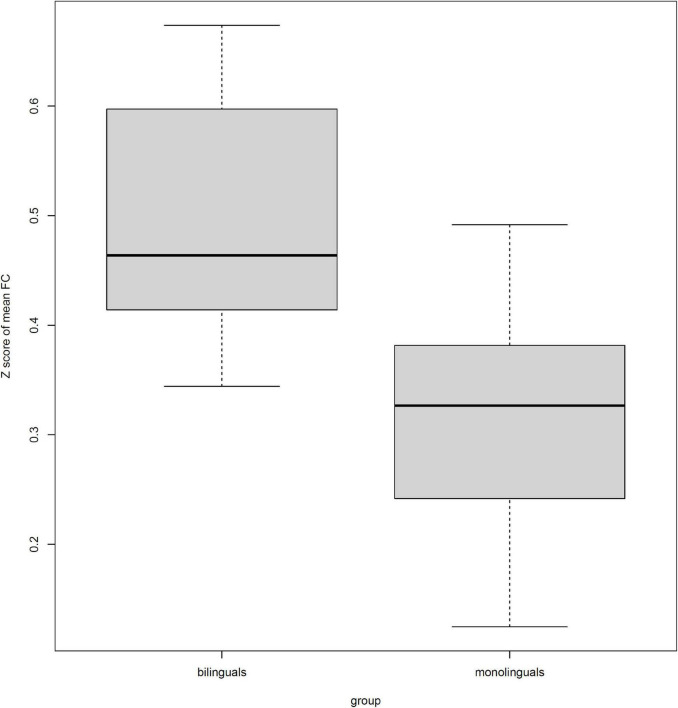
The differences in the mean of *Z* scored functional connectivity (FC) within the identified subnetwork between bilinguals and monolinguals.

Table 4 also shows that all 10 connections were stronger in bilinguals than monolinguals. The mean *Z* scores of functional connectivity in the identified subnetwork exhibiting group-wise differences revealed group differences between the bilinguals and monolinguals (0.44 ± 0.12 vs. 0.31 ± 0.10; *F* = *21.48*, *P* < 0.001) (Figure 2).

### Relationship Between Functional Connectivity Strength and Executive Function

**FIGURE 3:**
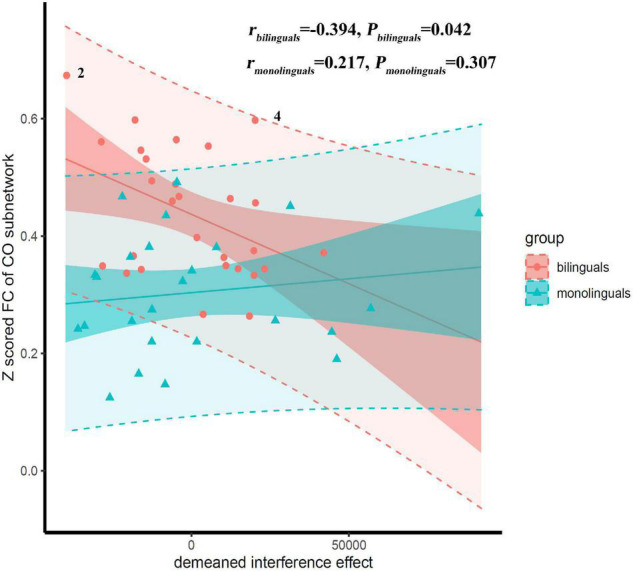
Scatter plot with regression lines showing correlations of the interference effect vs. mean *Z* scored functional connectivity (FC) of the cingulo-opercular (CO) subnetwork. The low-transparency shade represented 95% confidence interval and the high-transparency shade represented 95% prediction interval. There was a significant interaction effect between the group and the interference effect (*P* = 0.033). Correlations were significant only in the bilinguals, but not in the monolinguals. Suspected outliers were marked, the results of correlation analysis after removing the suspected outliers were reported in the Supplementary Table 2.

Correlations between the mean *Z* score of functional connectivity within the identified subnetwork and the inhibitory control, shift and working memory were estimated in the bilinguals and monolinguals (shown in Table 5). There was a significant interaction effect between the group and the interference effect (*P* = 0.033). Only in the bilinguals, were the mean *Z* scores of functional connectivity within the identified subnetwork (*r* = −0.394, *P* = 0.042) significantly negatively correlated with the interference effect (shown in Figure 3). Negative correlations indicate that bilingual individuals having better performance in the Stroop test had stronger functional connectivity of the CO subnetwork. However, this correlation did not survive the Bonferroni control (α/4 = 0.0125). In contrast, there was no significant correlation in the monolinguals (*r* = 0.217, *P* = 0.307). As for response inhibition, shift and working memory, there was no significant interaction effect of group nor correlation in the whole sample (all *P* > 0.05). To verify the robustness of the significant correlation, we retried the correlation analyses after deleting suspected outliers and the correlation was unchanged (see Supplementary Table 2).

**TABLE 5:** The correlation analysis between functional connectivity of CO subnetwork and EF.

Correlation, *r*(*P*)	Bilinguals	Monolinguals	*P*_interaction_
Functional connectivity-interference control	−**0.394(0.042)**	0.217(0.307)	0.033
Functional connectivity-response inhibition	−0.082(0.563)		0.258
Functional connectivity-shift	−0.132(0.351)		0.711
Functional connectivity-working memory	0.146(0.300)		0.349

*Significant results were bold. The P-values were indicated between parentheses. Pearson correlation analysis was performed in subgroups when P_interaction_ < 0.05, and in the whole samples when P_interaction_ > 0.05. CO, cingulo-opercular; EF, executive function.*

## Discussion

According to the dual-network model (Dosenbach et al., 2008; Gratton et al., 2018), EF networks include the FP and CO networks. In the current study, we used the NBS analysis to investigate differences in functional connectivity in the FP and CO brain networks between Cantonese-Mandarin bilingual and Mandarin monolingual college students. The results showed that the bilinguals had stronger functional connectivity than the monolinguals in the CO but not FP network. The identified CO subnetwork with group-wise differences contained 9 nodes and 10 edges, and the center node was the left mid-insula with a degree centrality of 5. There was a significant correlation between the Stroop effect and functional connectivity in the CO subnetwork in bilingual but not monolingual groups.

First, we found that compared to the Mandarin monolinguals, the Cantonese-Mandarin bilinguals had stronger functional connectivity in a subnetwork located in the CO network of the EF networks. The identified subnetwork referred to as the CO subnetwork consisted of 9 nodes and 10 edges, including left right dACC-left mid-insula- -bilateral basal ganglia-left angular gyrus/-bilateral thalamus, and the insula and basal ganglia constitute the core area of the CO network. According to the dual-network model, the CO network includes the anterior insula, basal ganglia and dACC (Dosenbach et al., 2008). Using fMRI, Marian et al. (2014) and Abutalebi et al. (2012) found that the activity in the ACC during EF tasks was lower in bilinguals than in monolinguals. In addition, a few structural fMRI studies indicated that the gray matter volume of the dACC (Abutalebi et al., 2012) and basal ganglia (Krizman et al., 2012, 2014) was larger in the bilinguals than in the monolinguals. Some studies have shown that the ACC and insula could downregulate the activities of the default brain network (DMN) and other internal processes unrelated to tasks to allocate cognitive resources to current external tasks (Menon and Uddin, 2010; Goulden et al., 2014). The basal ganglia are a group of interconnected gray matter nuclei located in the middle of the brain and control the signal inputs from the thalamus to the frontal lobes by balancing the inhibitory and excitatory signals transmitted by parallel pathways. In the bilingual brain, the basal ganglia controls which language to use (Stocco et al., 2014). The thalamus plays an important role in the competition of vocabulary and semantic representation in the process of bilingual language production due to its extensive connections with the lIFG and basal ganglia (Abutalebi and Green, 2016). In addition, the angular gyrus (Della Rosa et al., 2013; Seghier, 2013) and temporal (Friederici and Gierhan, 2013; Grant et al., 2015) also play important roles in the cognitive control of language.

The CO network is considered as a core system involved in cognitive control including maintaining task sets (Dosenbach et al., 2008), detection of errors and interference (Dosenbach et al., 2006; Seeley et al., 2007), and inhibitory control (Swick et al., 2011). We speculated that as logographic languages, Cantonese and Mandarin share the same writing system, so Cantonese-Mandarin bilinguals may need stronger EF to inhibit interference of the non-target language in daily communication and learning, regardless of which language is used. In addition, in mainland China, Mandarin and Cantonese enjoy strict functional separation (Ng and Zhao, 2014). The Cantonese-Mandarin bilinguals normally use Mandarin on formal occasion such as school and office, and Cantonese at home. According to the adaptive control hypothesis (Green and Abutalebi, 2013), such context is named single language context. The adaptive control hypothesis claimed that bilinguals in single language context need to establish and stabilize a control state, in which the interference from non-target language should be suppressed, while the response inhibition is less required. This was supported by a recent study (Lai and O’Brien, 2020). The long-term experience of Cantonese-Mandarin bilingualism inevitably shapes the EF networks related to inhibition, especially the interference control component, which leads to the stronger functional connectivity of the CO subnetwork of Cantonese-Mandarin bilinguals than that of the Mandarin monolinguals. Our speculation was supported by our correlation analysis. The classic Stroop test was used to measure the interference control, a component of inhibition control (Friedman and Miyake, 2017). Our correlation analysis showed that there was a significant correlation between the interference control and the strength of the functional connectivity within the CO subnetwork in Cantonese-Mandarin bilinguals; that is, bilinguals with stronger EF showed stronger functional connectivity within the CO subnetwork. Moreover, there was a significant interaction effect between the group and the interference control and this correlation did not exist in the Mandarin monolingual college student group. These results suggested that Cantonese-Mandarin bilinguals depend on this subnetwork more than monolinguals. However, this correlation did not survive the Bonferroni control, which indicated this correlation may be influenced by the limited sample size or some other factors. In future research, we would identify this association in a larger sample. No significant correlation was found between the functional connections within the CO subnetwork and response inhibition, another component of inhibition control. The absence of significance might be related to the fact that Cantonese-Mandarin bilinguals rely more on interference control but not response inhibition in a single language context.

In addition, though the CO network was also suggested to participate in working memory processing, no significant correlation was found between the functional connections within the CO subnetwork and working memory. A recent working memory model claims that working memory involves many basic cognitive processes including selective attention, inhibition and etc. (Eriksson et al., 2015). In fact, the CO network gets involved in working memory through cognitive control (Wallis et al., 2015). Grundy and Timmer (2016) suggested that the effect of bilingualism on working memory might be an indirect result of the high demand for attention and inhibition during language selection. Besides, their meta-analysis (Grundy and Timmer, 2016) showed that the effect of bilingualism on working memory was the weakest in young adults compared to the other age groups. Thus, the relationship between the CO subnetwork with group-wise differences and working memory may be indirect and weak, which is hard to be observed in a small sample of young adults.

Interestingly, in the current study, no significant difference in functional connectivity within the FP network was found between Cantonese-Mandarin bilinguals and Mandarin monolinguals. In contrast to our study, Grady et al. (2015) found that bilinguals had stronger functional connectivity than monolinguals in the FP network, while no difference was found in the CO network. The FP network includes dorsolateral and inferior frontal regions, as well as the inferior parietal lobes, which act to initiate and adjust control (Cole and Schneider, 2007). We speculated that there were 3 possible reasons for our results: first, the bilingual population in Grady’s study comprised elderly individuals with decreased cognitive flexibility, and bilinguals are likely to maintain cognitive flexibility, thus showing stronger connectivity in the FP brain network. However, the subjects recruited in our study were young adults (college students), who were presumably at the peak of their cognitive flexibility (Diamond, 2013), thus the effect of bilingualism on the FP brain network was not remarkable. Second, there might be a relationship with the language interactional contexts for bilingual speakers. The bilinguals of Grady’s study lived in Canada with a rich linguistic diversity, and need to switch from two languages frequently in daily communication situations. However, as mentioned above, the language interactional context for Cantonese-Mandarin bilinguals in our study belongs to the single language context, in which bilinguals use two languages in distinct contexts, while that for bilinguals in Grady’s belongs to the dual language context (Green and Abutalebi, 2013). Bilinguals in the single language context rely more on the goal maintenance and interference control which were related to the CO network, while bilinguals in the dual language context rely more on the ability of shift which was related to the FP network (Green and Abutalebi, 2013). Finally, considering the relatively limited sample size of our study, we caution that the negative result in FP network should be taken prudently.

Finally, we found that there was no significant difference in behavioral performance of EF, namely, inhibitory control, shift and working memory between the two groups. This may make the functional connection differences within the CO subnetwork confusing. We tend to further clarify the relationship between the MRI results and behavioral results here. The absence of significance in comparison of EF performance was always reported, especially in the population of young adults (Bialystok et al., 2005; Abutalebi et al., 2012; Kousaie and Phillips, 2012; Paap and Greenberg, 2013). The possible explanation was that the bilingualism effect on EF was subtle in young adults because their EFs are at the peak. However, no difference in the behavioral performance of EF doesn’t mean the same neural mechanism underlying EF. Both Rodriguez-Pujadas et al. (2013) and Ansaldo et al. (2015) didn’t observe significant difference between bilinguals and monolinguals in EF performance, but they reported different brain activation between the two groups during EF tasks. The same phenomenon has been seen in electrophysiological studies on bilinguals’ EF (Kousaie and Phillips, 2012; Wu et al., 2016): no difference was found in the behavioral performance of EF, but the event-related potentials showed different patterns between bilinguals and monolinguals during EF tasks. Cespón and Carreiras (2020) claimed that neurophysiological techniques are potentially more sensitive than behavioral measures on detecting differences in bilinguals’ EF. These studies suggest that although there is no difference in the performance of EF between bilingual and monolingual college students, their EF depends on different brain mechanisms. Our results of correlation analysis could also serve as evidence. The significant correlation between the interference control and functional connectivity within the CO subnetwork exists only in the bilingual group. The interaction effect between the group and the interference control is significant (*P*_interaction_ = 0.03), which supports differences in the neural mechanisms of EF between monolinguals and bilinguals.

### Limitations

We acknowledged that there are some limitations in the current study. First, the sample size was small, which may have affected the stability of the results. Chen et al. (2018) tested the sample size effect on the reproducibility of resting-state fMRI metrics and observed that the positive predictive value tended to stabilize when the sample size reaches a group of 40 subjects (80 subjects in total). In empiricism, the group differences were comparable and the results were reliable if the sample size is above 20 per group (Thirion et al., 2007; Goulden et al., 2012; Cao et al., 2014; Xu et al., 2015; Jung et al., 2018). As to the current study, according to the two groups’ *Z* scored functional connectivity within the CO subnetwork, the effect size (Cohen’s *d*) is calculated to be 1.00. We calculated the power of the current study by setting *d* = 1.00, α = 0.005(after NBS correction), tails = 2, sample size per group = 31, and the test = two-sample independent *t*-test in G-power (Faul et al., 2007), and we got a power of 0.84 for the current study. It is higher than the usual appropriate power of 0.8. The power of our study indicates the sample size of 31 per group in our study could be acceptable. However, it is worth note the power was computed *post hoc*, and resting-state fMRI researches on bilingualism with a larger sample size are still needed in the future. Second, all the participants recruited in our study were bilingual individuals, as college students in China must learn English as their second language in class. Learning second language might have attenuated the potential difference between the Mandarin group and Cantonese-Mandarin group in EF. However, English proficiency was matched between the two groups in the current study (see Table 1).

## Conclusion

Long-term bilingual experience using two similar logographic languages was associated with functional connectivity within the CO subnetwork but not in the FP network underlying EF, which might be related to inhibitory control.

## Data Availability Statement

The original contributions presented in the study are included in the article/Supplementary Material, further inquiries can be directed to the corresponding author.

## Ethics Statement

The studies involving human participants were reviewed and approved by the Medical Ethics Committee, Sun Yat-sen University (L2016) No. 036. The patients/participants provided their written informed consent to participate in this study.

## Author Contributions

MF, QW, YW, and XL designed the research. LC, XX, XF, JM, YW, NP, and ZY collected the data. LC, XX, XF, and JM analyzed the data and interpreted the results. LC and XX wrote the manuscript. XL developed the idea, organized and supervised the whole study, and the manuscript. All authors discussed the results and revised the manuscript.

## Conflict of Interest

The authors declare that the research was conducted in the absence of any commercial or financial relationships that could be construed as a potential conflict of interest.

## Publisher’s Note

All claims expressed in this article are solely those of the authors and do not necessarily represent those of their affiliated organizations, or those of the publisher, the editors and the reviewers. Any product that may be evaluated in this article, or claim that may be made by its manufacturer, is not guaranteed or endorsed by the publisher.

## Footnotes

1
https://www.ethnologue.com/language/zho

2
http://rfmri.org/DPARSF

3
http://www.fil.ion.ucl.ac.uk/spm

4
http://rfmri.org/DPABI

5
http://www.restfmri.net/forum/DPARSF

6
http://www.nitrc.org/projects/bnv/

## Supplementary Material

Ensure that any supplementary material is correctly published at this link: https://www.frontiersin.org/articles/10.3389/fnhum.2021.748919/full#supplementary-material

## Abbreviations

EF: executive function
lIFG: left inferior frontal gyrus
vmPFC: ventral medial prefrontal cortex
fMRI: functional magnetic resonance imaging
FPN: frontal-parietal network
CON: cingulo-opercular network
dACC: dorsal anterior cingulate cortex
NBS: network-based statistics
LBSQ: Language and Social Background Questionnaire
IQ: intelligence quotient
FOV: field of view
FD: frame-wise displacement
RT: reaction time
vFC: ventral frontal cortex.
