# Supplementary material for: Differences in Brain Functional Networks of Executive Function Between Cantonese-Mandarin Bilinguals and Mandarin Monolinguals
Source: Front Hum Neurosci. 2021 Nov 18;15:748919. doi: 10.3389/fnhum.2021.748919 (PMC8638783; doi:10.3389/fnhum.2021.748919)
Supplement: Supplementary file 1 [file Data_Sheet_1.docx]

# Supplementary materials

## 1 Executive function test materials

**A**

**B**

**C**


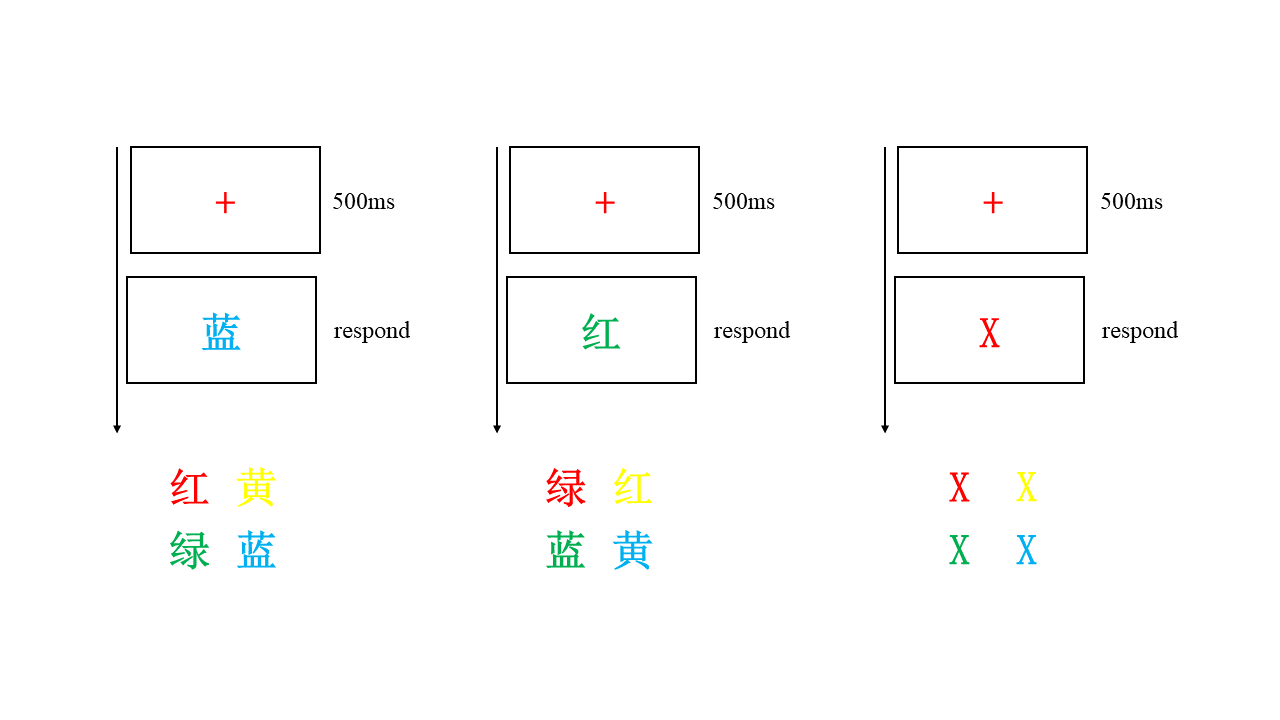


**Supplemental Figure 1. The trail design of the Stroop Color and Word Test. A represents a trial in congruent condition; B represents a trail in incongruent condition; C represents a trail in neutral condition.**

In Stroop test, subjects were asked to identify the color of the font presented by pressing the corresponding key. Four labels in the colors of red, yellow, blue, green were successively stuck onto the four keys of “F”, “G”, “H”, “J” on the keyboard. In each trail of Stroop test, a red fixation was first presented for 500ms, and then a colored character was presented waiting for the response. The response interval duration was not limited. The stimulus materials were below the relevant trail designs. There were 72 trails in congruent condition, 24 trails in incongruent condition and 24 trails in neutral condition. The trails were performed randomly.


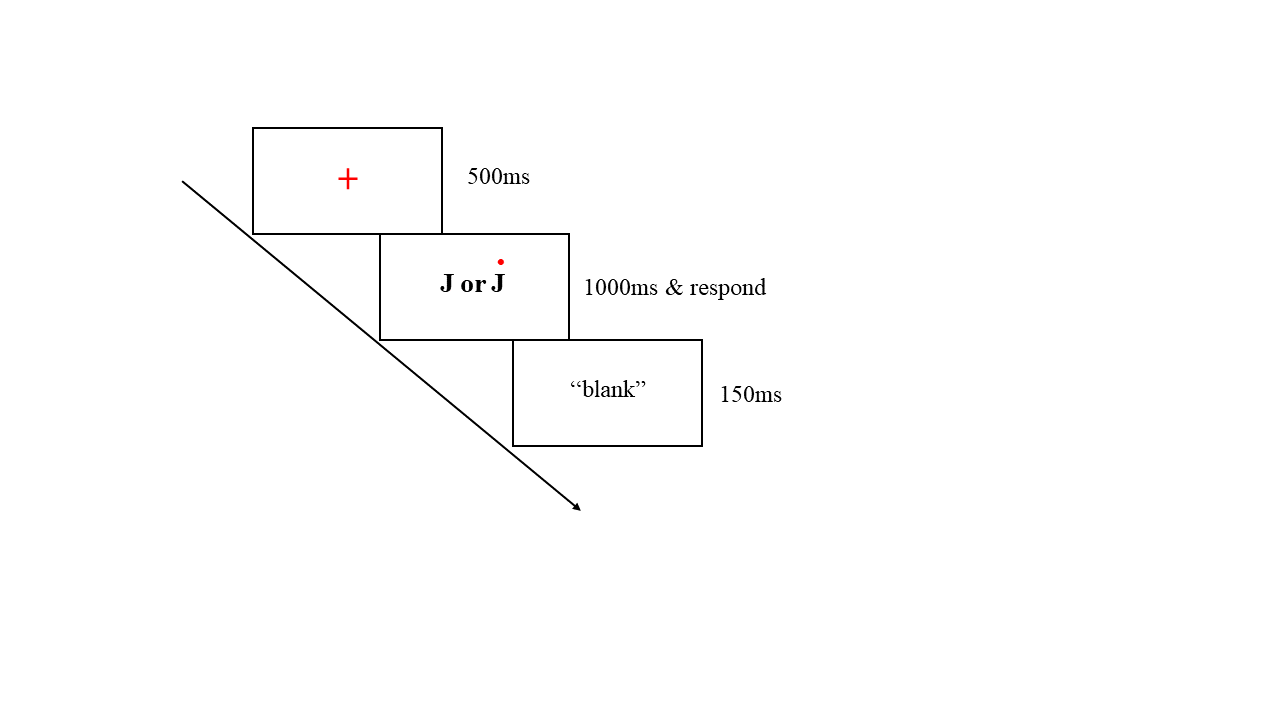


**Supplemental Figure 2. The trail design of the go/no-go paradigm.**

In the go/no-go paradigm, the stimulus of a capital letter (F or J) was presented and subjects were asked to press the corresponding key (“K” or “J”) on the keyboard, but withhold the response when a red dot appeared above the letter. In each trail, a red fixation was presented first for 500ms. Then, the stimulus material was presented for a maximum of 1000ms and meanwhile subjects should choose to respond or not. Before the next trail, a blank interval would last for 150ms after the representation of stimulus. A total of 60 trails were used in the formal test, 30% of which were no-go trails.


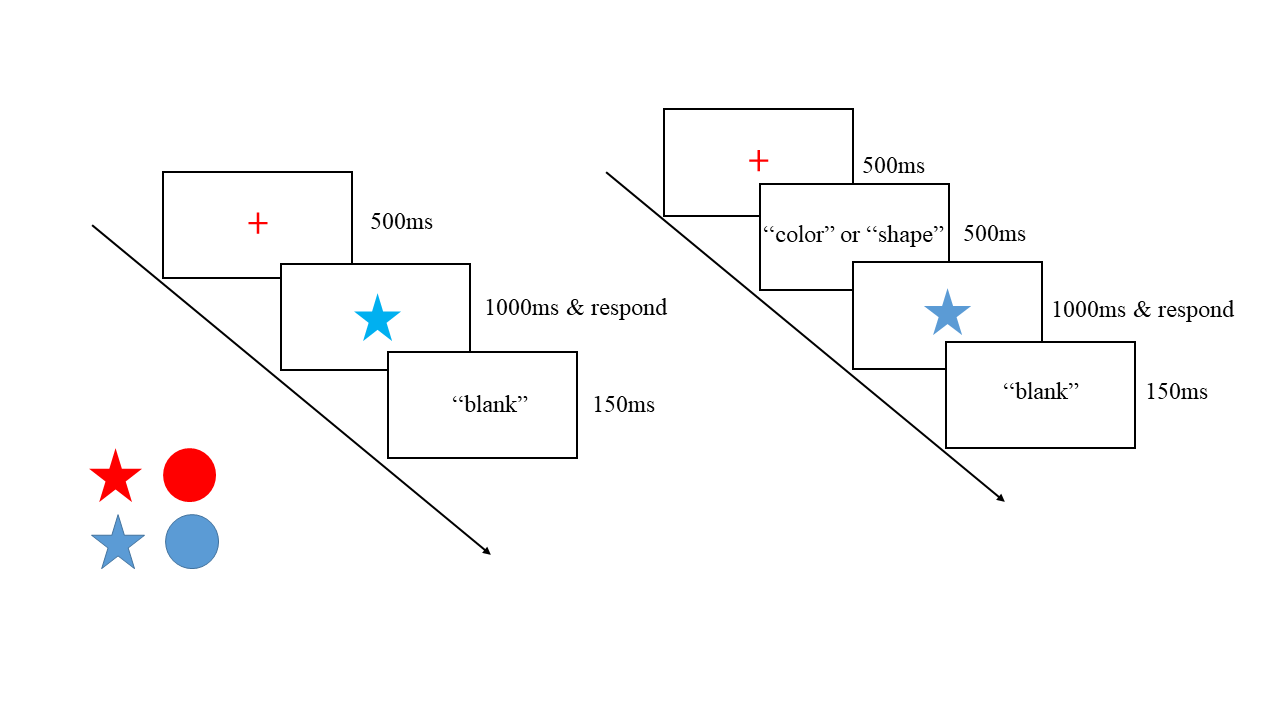


**C**

**B**

**A**

**Supplemental Figure 3. The trail design of the color-shape switch paradigm. A represents the single-task trial; B represents the mixed-task trial; C the stimulus materials.**

The subjects were asked to judge either the color or shape of the stimulus by press the corresponded keys on the keyboard in the color-shape switch paradigm. “F” corresponded to “blue” and “circle”, while “J” corresponded to “red” and “star”. In the first two blocks of single-task trials, the instruction was told to subjects first. A fixation was first presented for 500ms. Then the stimulus was presented for a maximum of 1000ms and meanwhile subjects were asked to respond. Before the next trail, a blank interval would last for 150ms after the representation of stimulus. In the third block of mixed-task trials, the hint words of instructions would be presented for 500ms before the stimulus.

## 2 Node definition for network construction

**Supplemental Table 1. Regions and MNI coordinates of the nodes constituting the CO and FP networks** (refer to *Dosenbach et al., 2010*).

|  |  |  |  | **MNI-coordinates** | | |
| --- | --- | --- | --- | --- | --- | --- |
| **Labels** | **Regions** | **Hemisphere** | **Subnetwork** | **x(mm)** | **y(mm)** | **z(mm)** |
| 1 | anterior prefrontal cortex | R | fronto-parietal | 29 | 57 | 18 |
| 2 | anterior prefrontal cortex | L | fronto-parietal | -29 | 57 | 10 |
| 3 | ventral anterior prefrontal cortex | R | fronto-parietal | 42 | 48 | -3 |
| 4 | ventral anterior prefrontal cortex | L | fronto-parietal | -43 | 47 | 2 |
| 5 | ventral lateral prefrontal cortex | R | fronto-parietal | 39 | 42 | 16 |
| 6 | dorsal lateral prefrontal cortex | R | fronto-parietal | 40 | 36 | 29 |
| 7 | anterior cingulate cortex | L | fronto-parietal | -1 | 28 | 40 |
| 8 | dorsal lateral prefrontal cortex | R | fronto-parietal | 46 | 28 | 31 |
| 9 | ventral prefrontal cortex | L | fronto-parietal | -52 | 28 | 17 |
| 10 | dorsal lateral prefrontal cortex | L | fronto-parietal | -44 | 27 | 33 |
| 11 | dorsal frontal cortex | R | fronto-parietal | 40 | 17 | 40 |
| 12 | dorsal frontal cortex | R | fronto-parietal | 44 | 8 | 34 |
| 13 | dorsal frontal cortex | L | fronto-parietal | -42 | 7 | 36 |
| 14 | inferior parietal lobe | L | fronto-parietal | -41 | -40 | 42 |
| 15 | inferior parietal lobe | R | fronto-parietal | 54 | -44 | 43 |
| 16 | posterior parietal | L | fronto-parietal | -35 | -46 | 48 |
| 17 | inferior parietal lobe | L | fronto-parietal | -48 | -47 | 49 |
| 18 | inferior parietal lobe | L | fronto-parietal | -53 | -50 | 39 |
| 19 | inferior parietal lobe | R | fronto-parietal | 44 | -52 | 47 |
| 20 | inferior parietal sulcus | L | fronto-parietal | -32 | -58 | 46 |
| 21 | inferior parietal sulcus | R | fronto-parietal | 32 | -59 | 41 |
| 1 | anterior prefrontal cortex | R | cingulo-opercular | 27 | 49 | 26 |
| 2 | ventral prefrontal cortex | R | cingulo-opercular | 34 | 32 | 7 |
| 3 | anterior cingulate cortex | L | cingulo-opercular | -2 | 30 | 27 |
| 4 | ventral frontal cortex | R | cingulo-opercular | 51 | 23 | 8 |
| 5 | anterior insula | R | cingulo-opercular | 38 | 21 | -1 |
| 6 | dorsal anterior cingulate cortex | R | cingulo-opercular | 9 | 20 | 34 |
| 7 | anterior insula | L | cingulo-opercular | -36 | 18 | 2 |
| 8 | basal ganglia | L | cingulo-opercular | -6 | 17 | 34 |
| 9 | medial frontal cortex |  | cingulo-opercular | 0 | 15 | 45 |
| 10 | ventral frontal cortex | L | cingulo-opercular | -46 | 10 | 14 |
| 11 | basal ganglia | L | cingulo-opercular | -20 | 6 | 7 |
| 12 | basal ganglia | R | cingulo-opercular | 14 | 6 | 7 |
| 13 | ventral frontal cortex | L | cingulo-opercular | -48 | 6 | 1 |
| 14 | middle insula | R | cingulo-opercular | 37 | -2 | -3 |
| 15 | thalamus | L | cingulo-opercular | -12 | -3 | 13 |
| 16 | thalamus | L | cingulo-opercular | -12 | -12 | 6 |
| 17 | thalamus | R | cingulo-opercular | 11 | -12 | 6 |
| 18 | middle insula | R | cingulo-opercular | 32 | -12 | 2 |
| 19 | middle insula | L | cingulo-opercular | -30 | -14 | 1 |
| 20 | basal ganglia | R | cingulo-opercular | 11 | -24 | 2 |
| 21 | posterior insula | L | cingulo-opercular | -30 | -28 | 9 |
| 22 | temporal | R | cingulo-opercular | 51 | -30 | 5 |
| 23 | posterior cingulate | L | cingulo-opercular | -4 | -31 | -4 |
| 24 | fusiform | R | cingulo-opercular | 54 | -31 | -18 |
| 25 | precuneus | R | cingulo-opercular | 8 | -40 | 50 |
| 26 | parietal | R | cingulo-opercular | 58 | -41 | 20 |
| 27 | temporal | R | cingulo-opercular | 43 | -43 | 8 |
| 28 | parietal | L | cingulo-opercular | -55 | -44 | 30 |
| 29 | superior temporal | R | cingulo-opercular | 42 | -46 | 21 |
| 30 | angular gyrus | L | cingulo-opercular | -41 | -47 | 29 |
| 31 | temporal | L | cingulo-opercular | -59 | -47 | 11 |
| 32 | temporal parietal junction | L | cingulo-opercular | -52 | -63 | 15 |

Note: This set of ROIs are from Dosenbach et al., 2010, Science. Each selected node was set to be a 5mm radius sphere and none of the nodes overlapped.

## 3 Outlier of the averaged functional connectivity


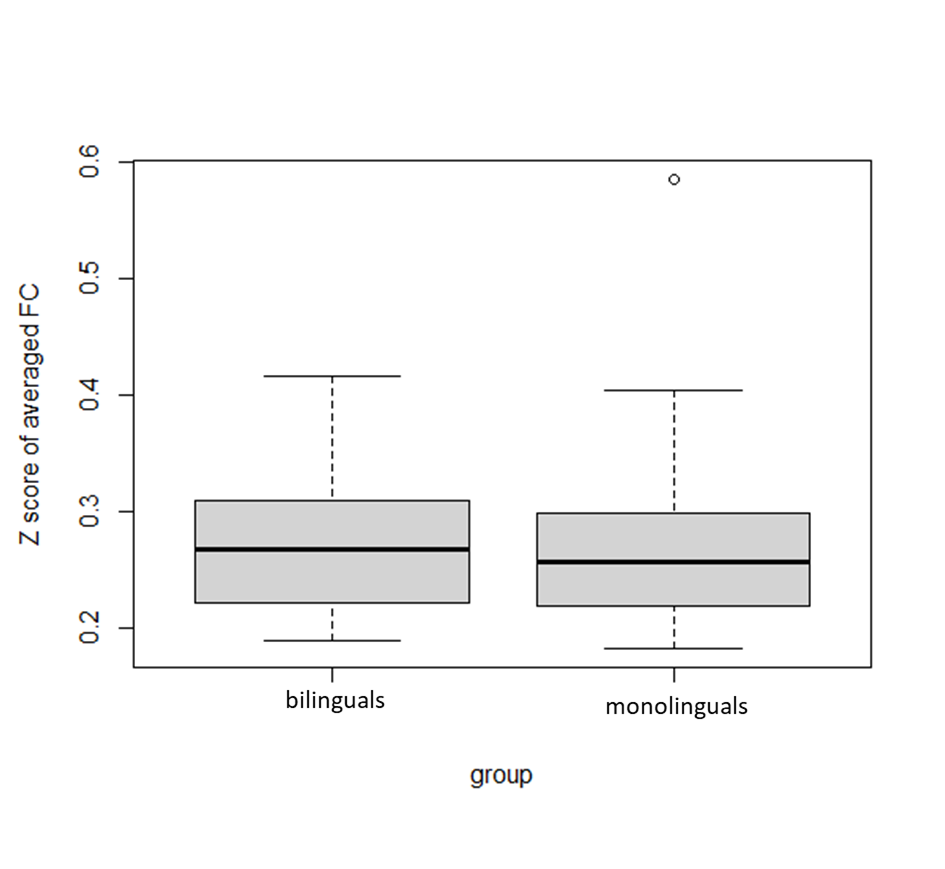


**Supplemental Figure 4.** Boxplot of averaged functional connectivity (FC) of the whole FC matrix (53×53). An outlier more than three interquartile ranges away from 75th percentile in the monolingual group was detected.

## 4 Correlation results after removing suspected outliers in the scatter plot

**Supplemental Table 2.** The correlations between FC of CO subnetwork and interference control in different subsets of data after removing suspected outliers.

| Deleted subject ID | Correlation, *r*(*P*) | Bilinguals | Monolinguals | *P*_interaction_ |
| --- | --- | --- | --- | --- |
| none | functional connectivity-interference control | -0.394(0.042) | 0.217(0.307) | 0.033 |
| 4 |  | **-0.474(0.015)** | 0.217(0.307) | 0.009 |
| 2, 4 |  | **-0.463(0.020)** | 0.217(0.307) | 0.016 |
